# Supplementary material for: The Control of the Crossover Localization in Allium
Source: Int J Mol Sci. 2023 Apr 11;24(8):7066. doi: 10.3390/ijms24087066 (PMC10138942; doi:10.3390/ijms24087066)
Supplement: Supplementary file 1 [file ijms-24-07066-s001.zip › SupplementaryMaterials.pdf]

Supporting Information

The Control of the Crossover Localization in Allium

SI Index: Supporting information for this manuscript includes the following items:

1) Figure S1. Trivalent scheme in F_1 triploid (*A. cepa* × *A. fistulosum*) hybrid ($2n = 3x = 16F+8C$)
2) Figure S2. Synaptonemal complex formation in *A. cepa*
3) Figure S3. Synaptonemal complex formation in *A. fistulosum*
4) Figure S4. Synaptonemal complex formation in F_1 diploid hybrid (*A. cepa* × *A. fistulosum*) ($2n = 2x = 8F+8C$)
5) Figure S5. Synaptonemal complex formation in F_1 triploid hybrid (*A. cepa* × *A. fistulosum*) ($2n = 3x = 16F+8C$)
6) Figure S6. Distribution of all possible combinations of MLH1/MUS81 mean foci number ratio in analyzed cells
7) Table S1. Pairwise comparisons of the mean foci number per cell for MLH1 and MUS81

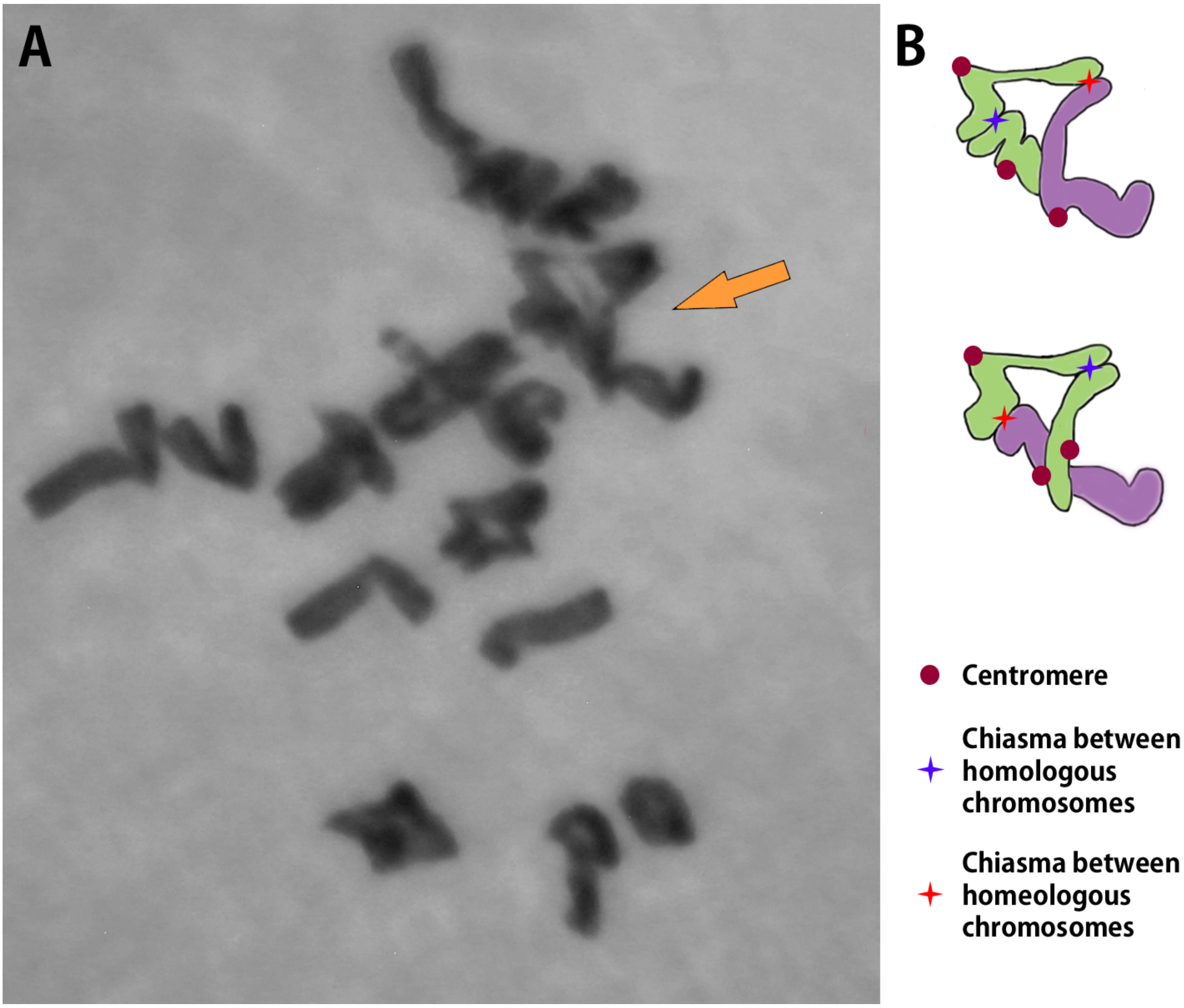

Figure S1. Meiotic metaphase I of F_1 triploid hybrid (*A. cepa* × *A. fistulosum*) ($2n = 3x = 16F+8C$) with trivalent (arrow) **(A)** and graphical interpretation of possible conformation of this trivalent **(B)**. According to the first trivalent scheme: the homologous chromosomes of *A. fistulosum* (green) formed a chiasma in the short arm, and the chromosome of *A. cepa* (violet) formed a chiasma with the chromosome of *A. fistolosum* in the long arm. According to the second scheme: the *A. cepa* and *A. fistulosum* chromosomes formed a chiasma in the short arm, while two homologous of *A. fistulosum* formed a chiasma in the long arm.

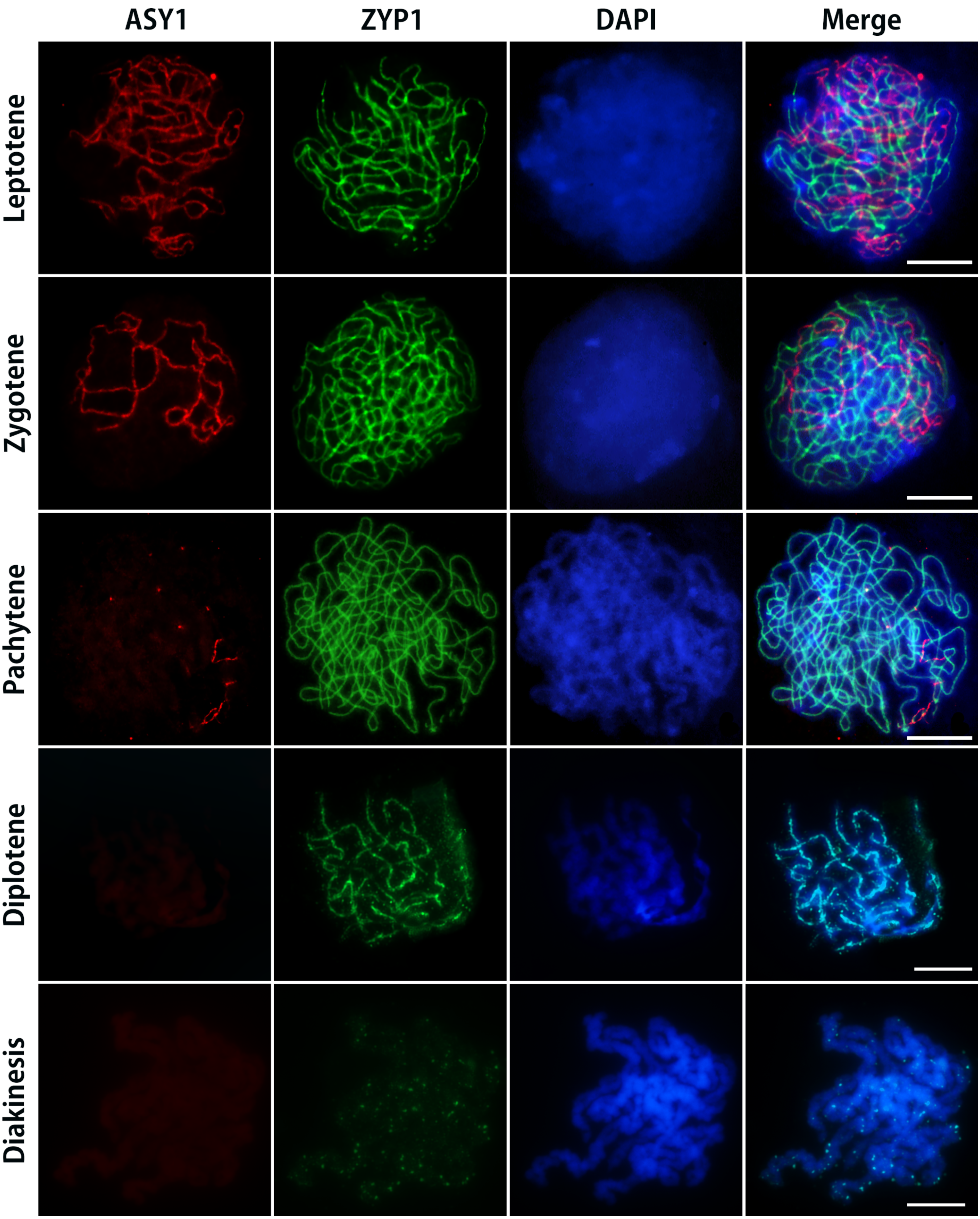

Figure S2. Synaptonemal complex formation in *A. cepa*. Nuclei at different meiotic stages immunostained with ASY1 (red) and ZYP1 (green) with DAPI counterstain (blue). Bar = 10 μm. At pachytene, all homologous are synapsed. At diakinesis, complete disassembly of the SC with ball-like structures of ZYP1 signals occurs.

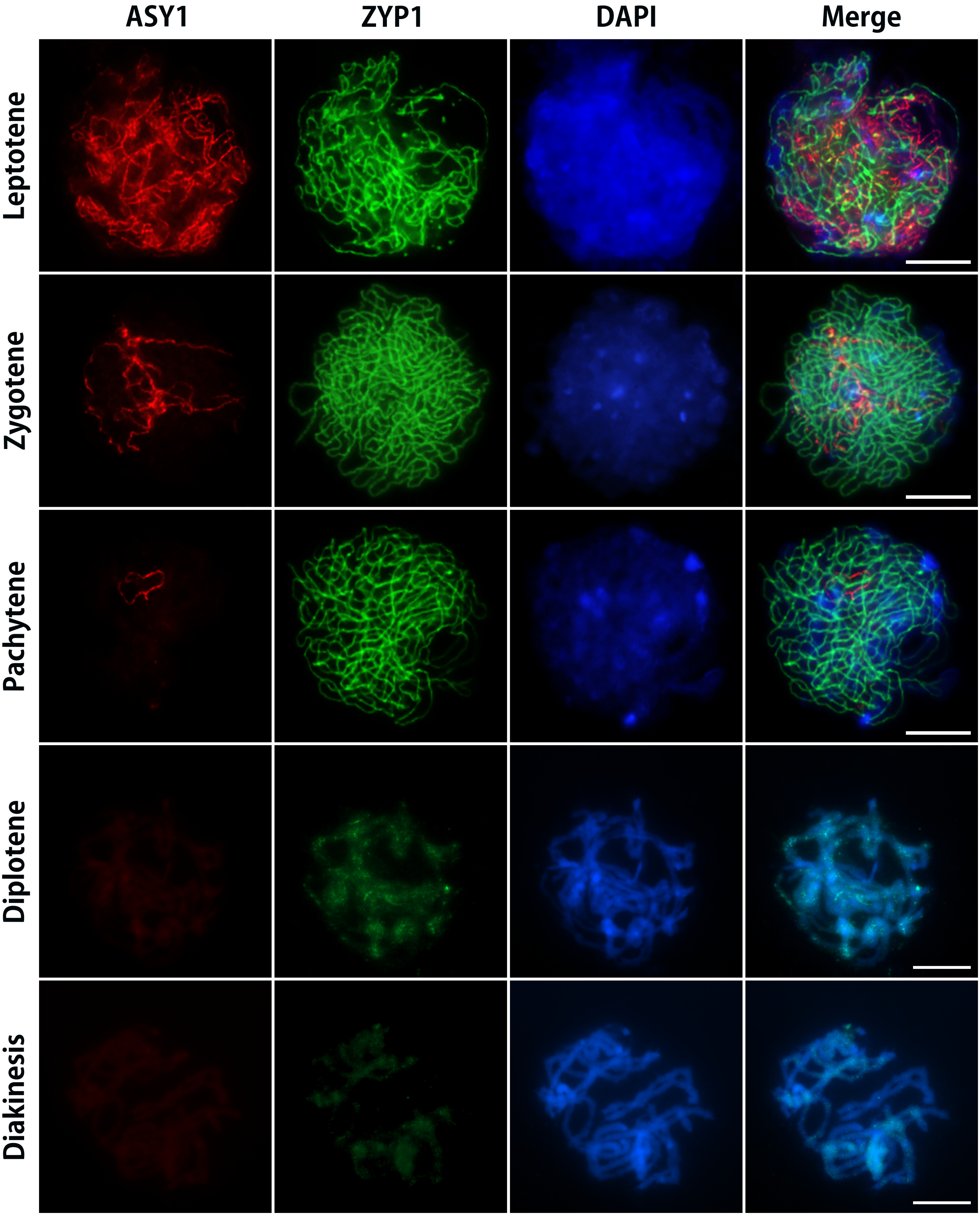

Figure S3. Synaptonemal complex formation in *A. fistulosum*. Nuclei at different meiotic stages immunostained with ASY1 (red) and ZYP1 (green) with DAPI counterstain (blue). Bar = 10 μm. At pachytene, all homologous are synapsed. At diakinesis, complete disassembly of the SC with ball-like structures of ZYP1 signals occurs.

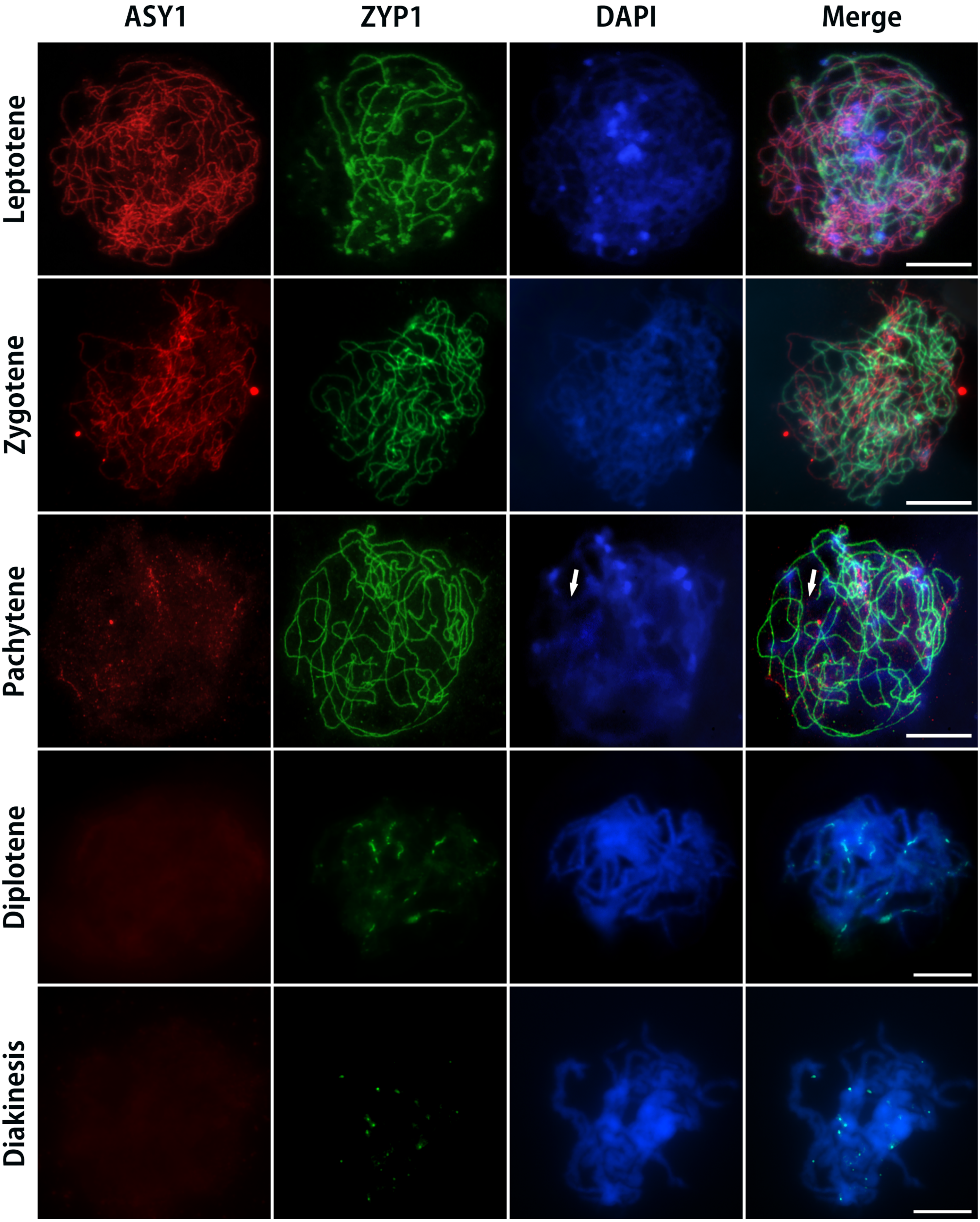

Figure S4. Synaptonemal complex formation in F_1 diploid hybrid (*A. cepa* × *A. fistulosum*) ($2n = 2x =$ 8F+8C). Nuclei at different meiotic stages immunostained with ASY1 (red) and ZYP1 (green) with DAPI counterstain (blue). Bar = 10 μm. From leptotene to pachytene, synapsis delay was occurring between the chromosomes of *A. cepa* and *A. fistulosum*. The arrow indicates non-synapsed region between homeologous chromosomes. At diakinesis, complete disassembly of the SC with ball-like structures of ZYP1 signals occurs.

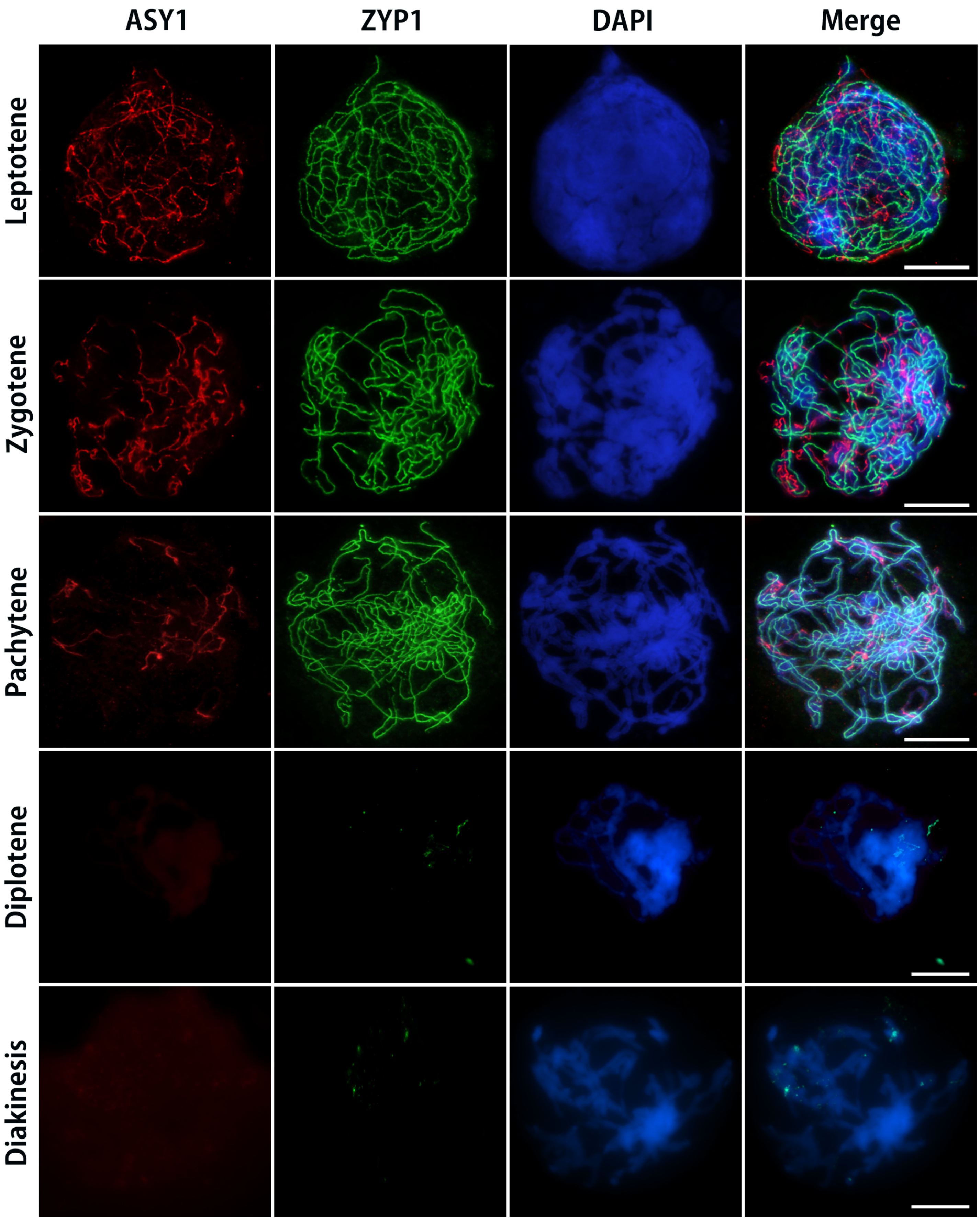

Figure S5. Synaptonemal complex formation in F_1 triploid hybrid (*A. cepa* × *A. fistulosum*) ($2n = 3x = 16F+8C$). Nuclei at different meiotic stages immunostained with ASY1 (red) and ZYP1 (green) with DAPI counterstain (blue). Bar = 10 μm. At pachytene, ASY1 long tracks can be explained by the presence of an unpaired set of *A. cepa* chromosomes, while short tracks of ASY1 probably derived from homoeologous chromosome pairing. At diakinesis, complete disassembly of the SC with ball-like structures of ZYP1 signals occurs.

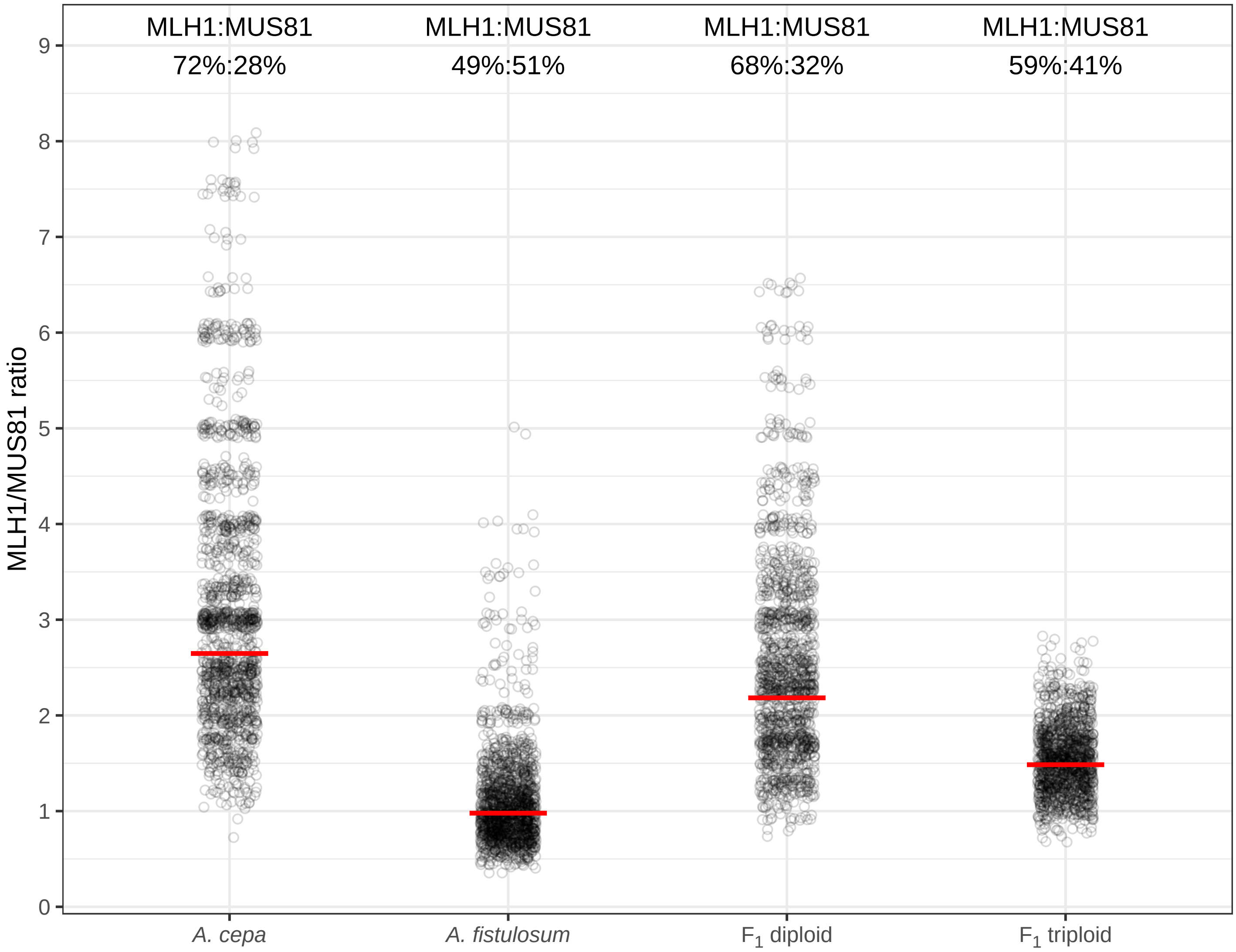

Figure S6. Distribution of MLH1/MUS81 ratio in all possible combinations of counted MLH1 and MUS81 foci per cell for *A. cepa*, *A. fistulosum*, F_1 (*A. cepa* × *A. fistulosum*) diploid and triploid hybrids. Red lines – weighted means with inverse weights in order to compensate effect from combinations with extreme MLH1/MUS81 ratios appearing because of artificial nature of generated population.

Table S1. Pairwise comparisons of MLH1 and MUS81 mean foci number per cell in *A. cepa*, *A. fistulosum*, F_1 (*A. cepa* × *A. fistulosum*) diploid and triploid hybrids.

a. MLH1

**Pairwise comparison**	**Observed mean foci per cell difference**	***P* value** [a]
A. cepa – *A. fistulosum*	4.6	<0.001
A. cepa – F_1 diploid hybrid [b]	2.6	<0.001
A. cepa – F_1 triploid hybrid [c]	2.6	<0.001
A. fistulosum – F_1 diploid hybrid	-2.0	<0.001
A. fistulosum – F_1 triploid hybrid	-2.0	<0.001
F_1 diploid hybrid – F_1 triploid hybrid	0.0	0.8

b. MUS81

**Pairwise comparison**	**Observed mean foci per cell difference**	***P* value** [a]
A. cepa – *A. fistulosum*	-2.4	<0.001
A. cepa – F_1 diploid hybrid	0.5	0.21
A. cepa – F_1 triploid hybrid	-1.6	<0.001
A. fistulosum – F_1 diploid hybrid	2.9	<0.001
A. fistulosum – F_1 triploid hybrid	0.8	0.01
F_1 diploid hybrid – F_1 triploid hybrid	-2.1	<0.001

[a] Wilcoxon test, FDR 5% corrected
[b] $2n = 2x = 8F+8C$
[c] $2n = 3x = 16F+8C$
